# Mode and Mechanism of Action of Omega-3 and Omega-6 Unsaturated Fatty Acids in Chronic Diseases

**DOI:** 10.3390/nu17091540

**Published:** 2025-04-30

**Authors:** Runcen Xu, Adrian J. Molenaar, Zhi Chen, Yuan Yuan

**Affiliations:** 1 Medical College, Yangzhou University, Yangzhou 225009, China; 18921879935@163.com; 2 Rumen Microbiology and Animal Nutrition and Physiology, Grasslands Research Centre, AgResearch, Fitzherbert, Palmerston North 4410, New Zealand; adrian.molenaar@agresearch.co.nz; 3 College of Animal Science and Technology, Yangzhou University, Yangzhou 225009, China; 4 School of Nursing and School of Public Health, Yangzhou University, Yangzhou 225009, China

**Keywords:** omega-3 fatty acids, omega-6 fatty acids, milk, neoplasms, linoleic acids, conjugated

## Abstract

Unsaturated fatty acids, particularly omega-3 and omega-6 polyunsaturated fatty acids, have garnered increasing scientific interest due to their therapeutic potential in chronic disease management. Dietary sources such as milk provide essential unsaturated fatty acids, including linoleic acid and α-linolenic acid. Current evidence indicates that these compounds and their derivatives regulate critical physiological processes, such as neurodevelopment, visual function, immune modulation, and cardiovascular homeostasis. Their multifunctional roles encompass the structural maintenance of biological membranes, cardioprotective effects, anti-inflammatory and anti-tumor activities, and metabolic regulation. However, despite established associations between unsaturated fatty acids and chronic diseases, the mechanistic contributions of omega-3 and omega-6 polyunsaturated fatty acids to complex neuropsychiatric disorders remain poorly characterized. Furthermore, the controversial role of omega-6 polyunsaturated fatty acids in chronic disease pathogenesis necessitates urgent clarification. This review systematically examines the structural properties, molecular mechanisms, and therapeutic applications of omega-3 and omega-6 polyunsaturated fatty acids in cardiovascular diseases, diabetes, cancer, dermatological conditions, neurodegenerative disorders, and depression. By integrating recent advances in dietary science, this work aims to address knowledge gaps in their neuropsychiatric implications and refine evidence-based strategies for chronic disease intervention through optimized nutritional approaches.

## Simple Summary

This study examines the roles of omega-3 and omega-6 polyunsaturated fatty acids (PUFAs) in chronic diseases, including cardiovascular disorders, diabetes, cancer, neurodegenerative conditions, and depression. Omega-3 PUFAs, primarily sourced from fatty fish, demonstrate protective effects by reducing inflammation, improving metabolic function, and lowering disease risk. They enhance cardiovascular health, mitigate insulin resistance, suppress tumor growth, and alleviate neuroinflammatory and depressive symptoms. Conversely, omega-6 PUFAs, prevalent in vegetable oils, are essential but may exacerbate inflammation when consumed excessively. The research underscores the critical balance between omega-6 and omega-3 intake, as their ratio significantly influences disease outcomes. Additionally, omega-3 PUFAs improve skin barrier integrity and reduce acne-related inflammation. By advocating dietary adjustments—such as prioritizing fish and minimizing processed oils—this work provides actionable insights to optimize PUFA intake, reduce chronic disease burdens, and advance public health. These findings bridge scientific evidence with practical dietary strategies, offering scalable solutions for global health improvement and healthcare cost reduction.

## 1. Introduction

Technological advancements have elevated global living standards and extended life expectancy. Nevertheless, population aging, escalating lifestyle stress, and the widespread adoption of unhealthy diets have precipitated a marked increase in subclinical health risks and chronic disease burdens, posing significant public health challenges. The pathogenesis and progression of these chronic diseases are associated with multifactorial determinants, including dietary patterns, epigenetic mechanisms, and gut microbiota interactions. While whole milk serves as a dietary source of modest quantities of ω-3 and ω-6 polyunsaturated fatty acids, its constituent exosomes may potentially exert deleterious effects by exacerbating disease risks, including type 2 diabetes mellitus, Parkinson’s disease, and various malignancies [1]. Furthermore, emerging evidence reveals a bidirectional interaction between epigenetic regulation and nutritional factors: specific genetic polymorphisms influence individual responsiveness to dietary constituents and nutrient requirements, while conversely, nutritional intake itself modulates gene expression patterns through epigenetic modifications [2]. Evidence indicates that unsaturated fatty acids critically modulate the onset, progression, and clinical outcomes of chronic conditions [3]. The structure of unsaturated fatty acids is very simple, consisting of only three elements: carbon, hydrogen, and oxygen. The only difference is the number of double bonds contained, and their locations are different. The first double bond in the chemical structure of omega-3 unsaturated fatty acids is located at the third carbon atom, while the first double bond of omega-6 unsaturated fatty acids is located at the sixth carbon atom. Accordingly, it is divided into two categories: omega-3 and omega-6 unsaturated fatty acids, which are also the two series of focus in this article. There are many research reports on omega-3 unsaturated fatty acids. Due to its excellent functions, such as regulating hemoesters, improving inflammation [4], reducing infection risk, improving cardiovascular function [5], and preventing diabetes [6], the application of omega-3 unsaturated fatty acids is quite widely used. Therefore, the research on omega-3 unsaturated fatty acids has been continuously expanded. However, although the research on omega-3 unsaturated fatty acids is so extensive, its role in neurological diseases such as Alzheimer’s disease (AD) and Parkinson’s disease (PD) and mental diseases such as depression still needs to be studied urgently. Although many speculations and assumptions have been made about the causes and mechanisms of these diseases, it is difficult to find some common and strong evidence to prove that these diseases have become pain points in drug development and clinical treatment.

Compared with those slow drug developments and painful clinical treatments, are omega-3 unsaturated fatty acids as nutritional interventions related to the occurrence and development of these diseases, and can it be used as the development direction of future disease research? This study will provide novel insights into previously investigated domains while elucidating the functional roles of newly identified mechanisms in areas where pathophysiological underpinnings remain poorly characterized. In contrast to the well-characterized roles of omega-3 unsaturated fatty acids, omega-6 counterparts remain poorly understood. Although omega-6 unsaturated fatty acids are not typically deficient in humans, their physiological impact—particularly their pro-inflammatory properties—remains contentious. Although omega-6 unsaturated fatty acids are extremely important fatty acids in the human body’s composition, not only does it receive less attention than omega-3 unsaturated fatty acids, but its therapeutic effect in chronic diseases such as cardiovascular diseases, diabetes, and cancer is highly questioned. Do omega-6 unsaturated fatty acids have a negative effect in these chronic diseases, and can they be used as part of nutritional intervention programs to have therapeutic effects on these diseases? Building upon contemporary research, this study not only conducts a comprehensive analysis of the respective roles of omega-3 and omega-6 polyunsaturated fatty acids in chronic diseases, but also examines their interconnected pathways in disease pathogenesis. The divergent biological significance of omega-3 and omega-6 unsaturated fatty acids arises from their distinct functional profiles, which are intrinsically linked to structural variations. By elucidating the relationships between chemical structure, biological function, and dietary sources, this work delineates the mechanisms underlying their roles in chronic diseases and proposes translational applications for public health.

## 2. Structural Properties, Metabolic Pathways, and Dietary Sources of Unsaturated Fatty Acids

### 2.1. Introduction to Unsaturated Fatty Acids

In the early 20th century, Thomas B. Osborne and Lafayette B. Mendel observed that rats fed a fat-free diet exhibited growth retardation, but growth resumed upon supplementation with ether extracts of butterfat, egg yolk, or cod liver oil, indicating the presence of an essential growth factor [7,8]. They erroneously attributed this factor to a “vitamin” rather than fatty acids [8,9]. Between 1929 and 1930, George Oswald Burr demonstrated through experimental studies that the critical lipid component required by rats was a fatty acid, not glycerol esters, and further identified linoleic acid as the essential fatty acid—a conclusion later corroborated by isotopic techniques. Subsequent research has elucidated the multifaceted health benefits of unsaturated fatty acids (e.g., EPA and DHA), including reducing cardiovascular disease risk via triglyceride regulation, anti-inflammatory effects, and vascular function improvement [10]; promoting neurodevelopmental and visual maturation in infants and children [11,12]; and ameliorating symptoms of neurodegenerative disorders (e.g., Alzheimer’s and Parkinson’s diseases) and psychiatric conditions [13].

### 2.2. Structure and Properties

The first double bond of omega-3 unsaturated fatty acid is located on the third carbon atom at the methyl end of the fatty acid chain, so it is named omega-3. It includes α-linolenic acid (ALA) (cis-9, cis-12, cis-15-octadecanotrienoic acid, 18:3), Stearidonic acid (SDA) (cis-6, cis-9, cis-12, cis-15-octadecanoic acid, 18:4), eicosapentadecanoic acid (EPA) (cis-5, cis-8, cis-11, cis-14, cis-17 -eicosapentaenoic acid, 20:5), docosapentaenoic acid (DPA) (cis-7, cis-10, cis-13, cis-16, cis-19-docosapentaenoic acid, 22:5), and docosahexaenoic acid (DHA) (cis-4, cis-7, cis-10, cis-13, cis-16, cis-19-docosahexaenoic acid, 22:6). Correspondingly, omega-6 unsaturated fatty acids that are the first double bonds located on the sixth carbon atom of the methyl terminus of the fatty acid, among which, the functions of linoleic acid (LA) (cis-9, cis-12-octadecanoic acid, 18:2) and arachidonic acid (AA/ARA) (cis-5, cis-8, cis-11, cis-14-eicosattetradecanoic acid, 20:4) are important. In many omega-3 unsaturated fatty acids, such as DHA, all double bonds exist in the cis configuration. However, trans-DHA can be synthesized by heating cis-linolenic acid to trans-linolenic acid and then extending it to trans-DHA [14]. Because the degree of unsaturation is so high, DHA is very susceptible to oxidation by free radicals [15], and according to Rabinovitch and Ripatti [16], polyunsaturated chains with double-bonded carbon atoms (the pattern found in DHA) separated by a methyl group have “maximum flexibility” and are extremely insensitive to temperature. This increased flexibility allows for easy folding into various secondary structures [17]. However, the flexibility of DHA may reduce the strength and increase the permeability of the lipid bilayer when incorporated [18]. On the other hand, molecular mechanics (MM) computer modeling studies performed on DHA have shown that polyunsaturated DHA chains are more rigid and ordered [19]. Conformational studies of small fragments similar to cis-DHA and other DHA analogs have been performed previously using MM methods [19]. They concluded that the planes of the double bonds are almost perpendicular to each other [16,19].

In animals and plants, these unsaturated fatty acids are mainly present in the form of triglycerides (TGs), followed by phospholipids (PLs), cholesterol esters (CEs), and fat-soluble vitamin esters (such as retinol palmitate and tocopherol acetate). Due to their different carriers, the absorption and utilization of fatty acids in different forms of existence are also different [20]. Unsaturated fatty acids become cholesterol, free fatty acids, monoglycerides, and other substances through the action of various lipases in the digestive tract. Emulsification of bile salts and phospholipids can promote its absorption and transport in the body [21]. However, its digestibility decreases with the increase of fatty acid chain length, from low to high, long-chain fatty acids, medium-chain fatty acids, and short-chain fatty acids [22]. Its existence form is also one of the factors that affect fatty acid digestibility. Although there are more fatty acids in the form of TGs, PLs exhibits higher utilization in vivo due to its excellent water dispersion and strong sensitivity to phospholipase [20]. In addition, differences in unsaturated fatty acids and carrier binding sites can also lead to different bioavailability. For example, DHA bound to the sn-1 position of a PL has higher bioavailability in the body. It can escape the hydrolysis of pancreatic phospholipase A2 (PLA2), and the fatty acids at the sn-2 position of glycerol will be released by PLA2. DHA is absorbed into lysophosphatidylcholine (LPC), and then converted to phosphatidylcholine (PC) by acyltransferase and then enters the lymph. The presence of DHA in plasma PCs can increase subsequent absorption of it by the brain [23]. Nevertheless, research in this area remains relatively underdeveloped, which could serve as a potential direction for future academic endeavors.

The role of serum albumin as the principal plasma carrier of free fatty acids in chronic diseases is mechanistically associated with its multifaceted ligand-binding properties, a functional characteristic attributable to the presence of key residues such as Cys34 and Lys59, which serve as strategic scaffolds for covalent drug modification [24]. Studies have demonstrated that unsaturated fatty acids (e.g., DHA, EPA) modulate drug efficacy via competitive binding at shared sites: Nuclear magnetic resonance experiments revealed significant competitive interactions between FFAs and warfarin (Sudlow site I, FA7) or ibuprofen (Sudlow site II, FA3/FA4) [25], evidenced by reductions in STD signals and specific intermolecular NOE effects, confirming their overlapping binding loci [26]. Molecular docking simulations integrated with X-ray crystallographic data demonstrate that FFAs adopt a dual anchoring mode at FA7 through carboxylate interactions with Arg-218 and His-242, exhibiting significant conformational flexibility through helical or hairpin folding to accommodate the binding pocket [26]. At FA4, FFAs establish dual-orientation binding via Arg-410/Tyr-411 or Ser-419/Thr-422 pairs, showing higher affinity compared to monounsaturated fatty acids [27]. Notably, low FFA concentrations induce HSA conformational changes that enhance warfarin binding, while elevated concentrations directly compete for FA7 occupancy, unveiling a lipid-mediated dynamic regulation mechanism. These findings elucidate the pharmacokinetic implications of FFA-albumin-mediated drug competition in chronic disease management (e.g., anticoagulant and anti-inflammatory therapies), providing molecular insights for optimizing drug-lipid interactions.

### 2.3. Metabolic Pathways

**Figure 1:**
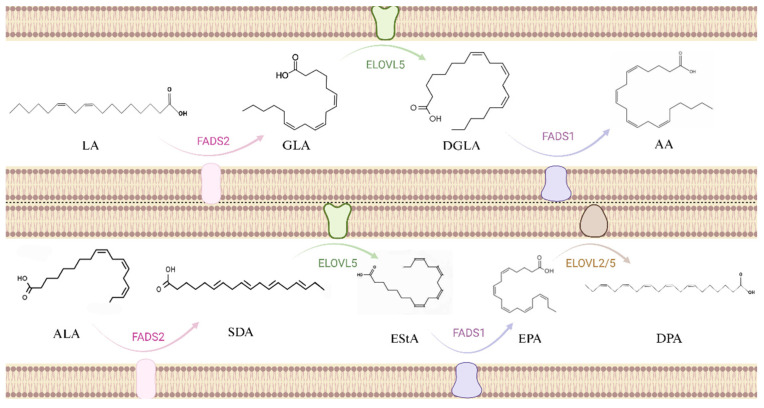
Metabolic pathways for synthesis of omega-6 unsaturated fatty acids and omega-3 unsaturated fatty acids. The abbreviation in the picture, Linoleic Acid: LA; Gamma-Linlenic Acid: GLA; Dihomo-Gamma-Linlenic: DGLA; Arachidonic Acid: AA; Alpha-Linolenic Acid: ALA; Stearidonic Acid: SDA; Eicosatetraenoic Acid: EStA; Eicosapentaenoic Acid: EPA; Docosapentaenoic Acid: DPA.

The substrate of the omega-6 unsaturated fatty acid synthesis pathway is LA, which synthesizes GLA, DGLA, and AA through fatty acid desaturase 1 (FADS1), elongase of very long chain fatty acids (ELOVL), and fatty acid desaturase 2 (FADS2), respectively [28]. Another path is to synthesize SDA, EPA, DPA, and DHA from ALA. The same enzyme was used in both paths (Figure 1). But because LA levels in human diets are usually higher than ALA levels, plasma and cellular levels of omega-6 unsaturated fatty acids tend to be higher than omega-3 unsaturated fatty acids. Therefore, omega-3 unsaturated fatty acids are more likely to be deficient than omega-6 unsaturated fatty acids. A study using stable isotopes showed that the efficiency of converting EPA, DPA, and DHA from ALA was 0.2%, 0.13%, and 0.05%, respectively [29]. This means that in addition to increasing dietary intake, improving metabolic conversion efficiency is also a solution. Desaturation has long been considered a rate-limiting step in the synthetic pathway [30], and these enzymes are encoded by the genes of fatty acid desaturases 1 and 2 located on chromosome 11. Studies have shown that the efficiency of several steps in the pathway, especially the desaturase step, is affected by genetic variation within the FADS cluster [31,32]. Therefore, the study of SNPs and epigenetic sites in FADS clusters may be one of the future directions to change the efficiency of metabolic synthesis.

### 2.4. Dietary Sources

Omega-6 unsaturated fatty acids are found in most crop seeds and vegetable oils, such as sesame oil, soybean oil, corn oil, peanut oil, safflower oil, and cottonseed oil. They are also found in seafood such as deep-sea fish and shellfish, such as tuna, yellow croaker, sardines, hairtails, etc. LA is an essential fatty acid in the human body and it plays an important role in reducing serum cholesterol [33]. The main metabolite of LA is arachidonic acid. Under the action of enzymes in vivo, intermediate products such as conjugated linoleic acid (CLA), gamma-linolenic acid (GLA) can be produced.

Compared with the widely distributed omega-6 unsaturated fatty acids, omega-3 unsaturated fatty acids have a risk of insufficient intake due to their limited sources [34]. Most algae are found to be rich in ALA, DHA, and EPA, as fish, shrimp, shellfish that feed on seaweed, and sea animals that feed on these animals and plants. ALA can be converted into DHA, DPA, EPA, etc., in the human body, but the conversion of Alpha-linolenic acid to EPA and DHA is very slow and the amount is small. In ordinary foods, ALA is relatively low, but in seaweed, deep-sea fish, and shellfish, it is rich in ALA and its derivatives. In addition, some terrestrial plant seeds also contain ALA, such as linseed oil, shepherd’s blue oil, and perilla seed oil. EPA mainly comes from cold-water fish, such as wild salmon, mackerel, sardines, and herbaceous fish. DPA is mainly found in the oils and fats of marine mammals and generally coexists with DHA. It is difficult to see purebred DPA in life. DHA is found in large quantities in fish oil in its natural form and also contains a portion in algae oil and egg yolks.

Despite over a century of comprehensive investigation into the structural characteristics of unsaturated fatty acids, substantial knowledge gaps persist regarding their functional roles and biological interaction mechanisms. Notably, critical aspects such as the precise compositional ratios among different unsaturated fatty acid subtypes remain understudied and warrant more systematic investigation.” to better convey the intended meaning while maintaining scientific accuracy. In addition, existing research focuses on the intake of single unsaturated fatty acids, but rarely considers their interactions. Future research needs to more comprehensively evaluate the impact of the proportion of different unsaturated fatty acids on the risk of chronic diseases in order to develop more scientific dietary recommendations. Today, due to its widespread natural distribution and its excellent role in a variety of chronic diseases, including cardiovascular disease, diabetes, cancer, skin diseases, neurodegenerative diseases, and depression, scientists use it as a nutritional intervention therapy that allows it to shine in clinical treatment.

## 3. Function and Mechanism of Unsaturated Fatty Acids in Diseases

### 3.1. Cardiovascular Disease

According to the World Health Organization, cardiovascular disease has become the leading cause of death worldwide, and low- and middle-income countries account for about 75% of them [35]. Genetics and epidemiology show that elevated plasma TG levels are an important risk factor for cardiovascular disease, and plasma TG is carried in the form of triglyceride rich lipoproteins (TGRL). TGRLs can lead to cardiovascular dysfunction and atherosclerosis in a variety of ways. Under inflammatory conditions, increased vascular permeability makes TGRL easy to pass through blood vessels, and dysfunctional endothelial cells express adhesion molecules (such as VCAM-1), recruiting mononuclear-macrophages. Macrophages absorb TGRLs through phagocytosis and then convert them into foam cells. In addition, the protein ApoC3, associated with TGRLs, has the activity of inhibiting lipase, which is also associated with an increased risk of CV [36]. Omega-3 unsaturated fatty acids are closely related to reducing the risk of cardiovascular disease, such as reducing TG levels.

Omega-3 unsaturated fatty acids can reduce TG levels through a variety of mechanisms. Omega-3 unsaturated fatty acids can increase the rate of fatty acid beta oxidation and inhibit acyl CoA [37]. The triglyceride-lowering effects of omega-3 unsaturated fatty acids are mediated through the direct inhibition of hepatic diacylglycerol acyltransferase (DGAT), an enzyme that catalyzes the terminal step in triglyceride biosynthesis by converting diacylglycerol to triglyceride—a process critical for intestinal triglyceride absorption and fatty acyl-CoA metabolism. Additionally, these fatty acids suppress phosphatic acid phosphohydrolase, a rate-limiting enzyme in the glycerol-3-phosphate pathway that facilitates triglyceride synthesis through sequential acylation reactions [38]. Omega-3 unsaturated fatty acids have been shown to enhance the rate of fatty acid β-oxidation and suppress acyl-CoA synthetase activity, thereby contributing to the reduction of triglyceride (TG) levels [37]. In addition, omega-3 unsaturated fatty acids can also reduce fat production and subsequent VLDL production in the liver by inhibiting diacylglycerol acyltransferase (DGAT) [39,40]. Among them, EPA and DHA can also reduce ApoC3 levels and enhance lipoprotein lipase (LPL) activity, resulting in receptor-mediated endocytosis of lipoprotein particles. Although the mechanism by which EPA reduces TG is not yet fully defined, EPA is a powerful agonist of peroxisome proliferator-activated receptor (PPAR) compared to other omega-3 unsaturated fatty acids [41]. PPAR agonists are excellent methods to reduce TG, including the thiazolidinediones (PPARγ agonists) and fibrate (PPARα agonists) that are widely used today. In addition, vascular endothelial dysfunction also plays a decisive role in the occurrence, development, and prognosis of atherosclerosis. Studies have shown that omega-3 unsaturated fatty acid intake is related to improvements in endothelial function. Potential mechanisms by which omega-3 unsaturated fatty acids enhance endothelial function include reducing the production of inflammatory cytokines and increasing endothelial-dependent vasodilation by promoting the release of NO [42,43]

Since increased levels of omega-6 unsaturated fatty acids in the diet increase the production of prostaglandins 2 and leukotriene 4, and these two types of substances can play a powerful pro-inflammatory role, omega-6 unsaturated fatty acids may theoretically increase the risk of cardiovascular disease [44]. But this view is still controversial. As mentioned above, people have a tendency to eliminate omega-6 unsaturated fatty acids. But, a meta-analysis of observational studies showed that omega-6 unsaturated fatty acid intake was negatively correlated with cardiovascular disease risk [45]. The current view is that the ratio between omega-6 unsaturated fatty acids and omega-3 unsaturated fatty acids may be more important than the absolute intake of both. In addition, highly unsaturated fatty acids such as AA in omega-6 unsaturated fatty acids may increase lipoprotein’s sensitivity to oxidation, leading to the formation of atherosclerosis [46].

The incidence of cardiovascular disease is positively correlated with the blood lipid level, so the hypolipid-lowering effect of omega-3 unsaturated fatty acids can theoretically reduce its incidence to a certain extent. However, despite the effective use of a variety of blood lipid-lowering drugs to reduce blood lipid levels, cardiovascular disease remains one of the leading causes of death worldwide. Given that the risk factors for cardiovascular disease are not only lipid levels, the damage of vascular endothelial cells and the production of inflammatory factors can aggravate the progress of cardiovascular disease, and the damage of endothelial cells is often related to the production of local inflammatory factors. Therefore, in the treatment of cardiovascular diseases, the effect of unsaturated fatty acids on inflammatory factors should still be of concern. Compared with the direct anti-inflammatory effects of omega-3 unsaturated fatty acids, the metabolites of omega-6 unsaturated fatty acids have theoretically pro-inflammatory effects. However, in actual experiments, increasing the intake of omega-6 unsaturated fatty acids will not increase the concentration of many inflammatory markers [47]. It is speculated that it is related to the ratio of omega-3 unsaturated fatty acids and omega-6 unsaturated fatty acids. The author believes that this aspect may be explored. The study revealed that comparative analysis of erythrocyte membrane lipids between cardiovascular disease patients and healthy individuals using nuclear magnetic resonance (NMR)-based lipidomics technique demonstrated significant lipid abnormalities in patients. Specifically, there were pronounced elevations in cholesterol, sphingolipids, and saturated/monounsaturated fatty acids, coupled with reduced levels of phospholipids (glycerophospholipids and ether-linked glycerolipids) as well as unsaturated/polyunsaturated fatty acids. These lipid alterations exhibited gradient differences across mild, moderate, and severe subgroups stratified by degree of coronary artery stenosis, suggesting that lipid metabolism disturbances may play a crucial role in the progression of atherosclerosis [48].

### 3.2. Diabetes

Diabetes are divided into types 1 and 2. Type 1 diabetes is usually caused by damage to the immune system, and the insulin-producing cells are destroyed, the body no longer produces insulin or insufficient insulin is produced. Type 2 diabetes is caused by insulin, resistance in the patient’s body, where fat tissue, muscles, and liver cells no longer use insulin, and then the body gets the wrong instructions to secrete more insulin. Type 2 diabetes has also become one of the top ten major causes of death, so it is crucial to explore the prevention and treatment measures of the disease [49,50]. The global increase in type 2 diabetes can be attributed to inappropriate eating patterns and sedentary lifestyles [51]. Therefore, dietary strategies are crucial for diabetes control [52]. The study found that increased fish and seafood intake in northwestern Greenland was associated with a reduced risk of type 2 diabetes. As an important component of seafood fatty acids, omega-3 unsaturated fatty acids is speculated to be related to their effects [53]. Insulin resistance and β-cell dysfunction are the basis of the pathogenesis of type 2 diabetes, and the increase in free fatty acids (FFA) levels further promotes the occurrence and development of the disease.

**Figure 2:**
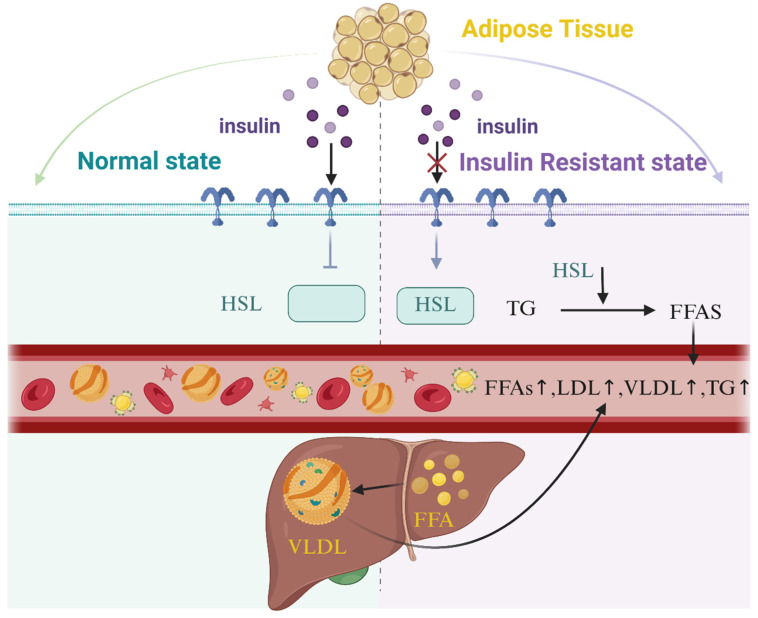
Relationship between elevated free fatty acids (FFAs) and insulin resistance: Insulin binding to receptors is blocked during insulin resistance, HSL is activated, and FFA levels in the liver are elevated. After some FFA in the liver is absorbed and lipidized, monoglycerides and FFA are formed under the action of LPL hydrolysis. This cycle continues to increase FFA in the blood.

Hormone-sensitive triglyceride lipase (HSL) is one of three lipases required for normal energy metabolism of adipose tissue. It is strictly controlled by insulin [54] and is inactivated when insulin binds to its receptor. In the insulin resistance state, insulin binding to its receptor is blocked, and the receptor activates HSL and hydrolyzes lipids such as triglycerides (TG), and the FFA levels released into the circulation of the liver are increased. After the liver absorbs and lipidizes part of FFA, it forms monoglycerides and FFA under the action of LPL hydrolysis. This circulatory process will also continue to increase FFA in the blood [55] (Figure 2). High TGs levels in the body will increase FFA levels, which will lead to the accumulation of DAG fatty acyl CoA and the increase of reactive oxygen species (ROS). Over time, mitochondria are lost, and leads to increased oxidative stress and insulin resistance [56]. Omega-3 unsaturated fatty acids can reduce the level of TGs in the body through the methods explained in the “Cardiovascular Disease” section, thereby reducing the risk of type 2 diabetes. In addition, omega-3 unsaturated fatty acids can also improve insulin resistance, reduce oxidative stress and anti-inflammatory effects through PPAR-α and PPAR-γ activation [57,58,59]. Enhancing the β oxidation of fatty acids and reducing muscle fat accumulation is also one of the methods to increase insulin sensitivity by affecting mitochondrial and endoplasmic reticulum functions [60].

There has been controversy over the association between omega-6 unsaturated fatty acids and diabetes. A meta-analysis showed that high dietary LA intake and increased LA concentrations in vivo were significantly associated with a lower risk of type 2 diabetes. In addition, the association between LA and reducing diabetes risk was also significant when 5.5–7.0% of energy intake came from LA [61]. Although there is a negative correlation between an LA diet and biomarker levels and diabetes risk, the mechanisms are unclear. Some possible explanations are as follows: elevated levels of unsaturated fats such as LA in the cell membrane may improve cell mobility and function, such as GLUT translocation, cellular signaling, ion permeability, and insulin receptor binding and affinity, which may lead to higher insulin sensitivity [62]. LA can also play a role in the regulation of genes such as Sterol regulatory element-binding protein-1 (SREBP1), which balances fatty acid synthesis and oxidation [63], a possible mechanism for LA to reduce liver fat content [64]. In addition, a diet with high levels of LA can effectively improve abdominal fat distribution and insulin sensitivity [65].

Some patients with type 2 diabetes have excessive insulin secretion, which leads to a certain extent to affect the efficacy after insulin use, so drug treatment is usually used. However, patients take medication for too long, which will cause complications such as hypoglycemia, obesity, and fatigue. As the disease progresses, patients will experience fat, protein, and carbohydrate disorders. During the treatment process, the patient’s physical and mental health cannot be protected. Therefore, omega-3 unsaturated fatty acids can avoid the occurrence of the above-mentioned drug complications by directly improving insulin resistance. The author believes that the role of unsaturated fatty acids in improving insulin resistance can be studied in depth, which can fundamentally treat type 2 diabetes. Furthermore, given the high prevalence of comorbid cardiovascular disease in diabetic populations, the established lipid-lowering effects of unsaturated fatty acids in cardiovascular risk management may concurrently offer synergistic benefits for diabetes prevention, thereby providing a dual therapeutic advantage through a single intervention. Although the mechanism of action of omega-6 unsaturated fatty acids in type 2 diabetes remains vague, their effects are significant [66]. The author believes that although experiments show that an LA diet has the effect of improving insulin sensitivity, the specific intake needs to be clear, and it is necessary to determine whether the intake is suitable for humans. Furthermore, since LA is a precursor of arachidonic acid (AA), which converts arachidonic acid into many pro-inflammatory metabolites [67], this raises concerns about increased LA intake. However, the author believes that this concern ignores arachidonic acid, which may also lead to an increase in other special anti-inflammatory molecules [68].

### 3.3. Cancer

It is well known that inflammation is a driving factor in cancer and can promote outbreaks of multiple tumor types [69,70], and the inflammatory microenvironment is an important part of all tumors. Some studies have shown that intake of omega-3 unsaturated fatty acids can reduce the risk of developing a variety of cancers, such as leukemia [71,72], breast cancer [73,74], colon cancer [75,76,77], prostate cancer [78,79], and melanoma [80]. Intake of omega-6 unsaturated fatty acids has a cancer-causing effect, which may be related to the increase in the proportion of arachidoid acid [81]. However, there are also studies that omega-6 unsaturated fatty acids can inhibit the growth of cancer cells by inducing ROS production and mitochondria damage [82]. Evidence indicates that a lower ω-6/ω-3 polyunsaturated fatty acid ratio is associated with upregulation of tumor suppressor SMAR1 expression and concurrent downregulation of the oncogenic factor MARBP Cux/CDP expression. Furthermore, in cells treated with low omega-6 unsaturated fatty acids/omega-3 unsaturated fatty acids, an increase in SMAR1 expression stimulates the expression of p21 protein that inhibits cell cycle progression [83]. DHA can selectively mediate the apoptosis of human MCF-7 breast cancer cells through caspase-8 activation [84]. Krill oil contains high concentrations of phospholipids, EPA, and DHA, and treatment with krill oil leads to a significant increase in mitochondrial membrane potential [85]. Due to changes in mitochondrial potentials, cells treated with EPA, DHA, and krill oil rich in these fatty acids result in an increase in ROS formation in colorectal cancer cell lines. The increase in ROS generation can stimulate the proapoptotic mechanism by changing the active form of caspase-3 and caspase-9 expression [85]. Mitochondria are also the source of NO. NO production is catalyzed by mitochondrial nitric oxide synthase (mtNOS) [86]. NO and inducible nitric oxide synthase (iNOS) promote cancer development. Peroxidation products of EPA and DHA reduce pro-inflammatory cytokine stimulation by inhibiting iNOS induction, thereby reducing NO production in hepatocytes. Preventing the production of NO is considered to be one of the effective indicators of anti-inflammatory effects [87]. Recently, in castration mice transplanted with TRAMP-C2 prostate tumor cells, a reduction in tumor growth was observed under the omega-3 unsaturated fatty acid diet due to increased cell levels of EPA and its oxidized derivatives F4-neuroprostaglandins (F4-NeuroPs) and resolvins, and reduced levels and content of the proangiogenic AA derivatives Prostaglandins 2 (PGE2) [88]. DHA is also effective in inhibiting the growth of IGROV-1 ovarian cancer cells. DHA treatment leads to phase G1 arrest and leads to downregulation of cyclins CDK4, CDK6, cyclin D1, and anti-apoptotic protein Mcl-1 [89].

**Figure 3:**
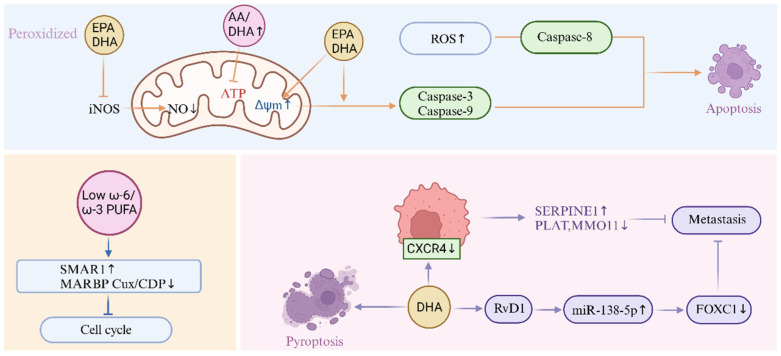
Effect of unsaturated fatty acids on cancer: EPA and DHA increase mitochondrial membrane potential (ΔΨm), peroxidized EPA and DHA inhibit iNOS, and then produce NO. The increase in the AA-DHA ratio inhibits ATP production; the low omega-6 unsaturated fatty acid/omega-3 unsaturated fatty acid ratio increases the expression of tumor suppressor SMAR1 and reduces the expression of tumor activator MARBPCux/CDP; metastasis is also regulated by unsaturated fatty acids, and induced apoptosis and pyroptosis through caspase pathway activation.

Cancer cell metastasis is the main cause of death in most cancers. Metastatic cell migration requires the upregulation of chemokine receptors, which act as molecular sensors for cell trafficking. CXCR4 is one of the chemokine receptors that are critical for chemotherapy of metastatic cells, and treatment of MDA-MB-231 breast cancer cells with DHA reduced the surface expression of CXCR4 and reduced CXCR4-mediated cell migration. In addition, DHA and its metabolite defiminant D1 (RvD1) also have a certain effect on the growth and invasion of cancer cells [90]. Figure 3 summarizes the role of unsaturated fatty acids in cancer. To sum up, as a type of disease that can cause serious consequences, early prevention is one of the most effective means to fight cancer. Nutritional intervention through the intake of unsaturated fatty acids has, therefore, become a hot research topic. Studies have shown that omega-3 unsaturated fatty acids play a significant role in the prevention and treatment of various cancers. The author believes that mitochondria are important metabolic and energy-supplying organs of cells. Future research can focus on the various effects of unsaturated fatty acids on mitochondrial components, such as the above mentioned promotion of ROS generation to promote regulating and inhibiting NO generation to prevent inflammation. In addition, the proportional relationship between omega-3 unsaturated fatty acids and omega-6 unsaturated fatty acids is also mentioned in the above “Cardiovascular Disease” section. Therefore, the author believes that the proportional relationship between the two in the diet should be of great significance to both normal people and patients, which can be the research direction for dietary intake of unsaturated fatty acids. DHA and EPA may also have the effect of increasing tumor sensitivity to radiotherapy while protecting skin tissue in non-radiative areas. Therefore, omega-3 unsaturated fatty acids can assist in radiotherapy, chemotherapy, and other interventions during the treatment of cancer patients to further strengthen the efficacy of cancer.

### 3.4. Skin Diseases

Since Burr first described the syndrome caused by a fat-loss diet in 1929, which mainly manifests as skin symptoms such as desquamation, hair loss, itching, and increased water loss, there has been a growing body of evidence that specific fats play a crucial role in skin structures [91,92]. In rodents, the problem of increased squamous skin and skin permeability can be restored by topical application of LA [93]. LA plays a role in maintaining skin barriers through different mechanisms. One mechanism is the activation of peroxisome proliferator-activated receptors (PPARs). PPARs are involved in a variety of pathways, including lipid metabolism, inflammation, keratinocyte differentiation, and permeability barrier homeostasis [94]. In an in vitro model of fetal skin development, the application of LA promotes the formation of skin barriers and significantly reduces trans epidermal water loss (TEWL) by activating PPAR-α [95]. Furthermore, LA can provide energy through beta oxidation, thereby promoting sebaceous gland synthesis of squalene and wax esters associated with skin barriers [96]. Acne is a chronic inflammatory skin disease that affects the hair follicles of the sebaceous glands and may form permanent scars. Therefore, adequate early treatment is necessary to prevent scarring and psychological sequelae [97].

The anti-inflammatory activity of omega-3 unsaturated fatty acids has been discovered for decades, and inflammation is one of the most important pathogenic factors of acne, so they have good results in the treatment in acne vulgaris. omega-3 unsaturated fatty acids, especially DHA, can inhibit the dimerization of TLR-1 and TLR-2 signaling [98]. *Propionibacterium acnes* increases the expression of TLRs on keratinocytes and macrophages, which leads to excessive proliferation and inflammatory responses of keratinocytes. *Propionibacterium acnes* induces monocyte TLR-2 activation and leads to the production of proinflammatory cytokines, including IL-1β, TNF-α, IL-8, and IL-12 [99]. Therefore, DHA and EPA, which inhibit the activation of TLR signaling pathways, may reduce inflammatory responses in acne patients.

**Figure 4:**
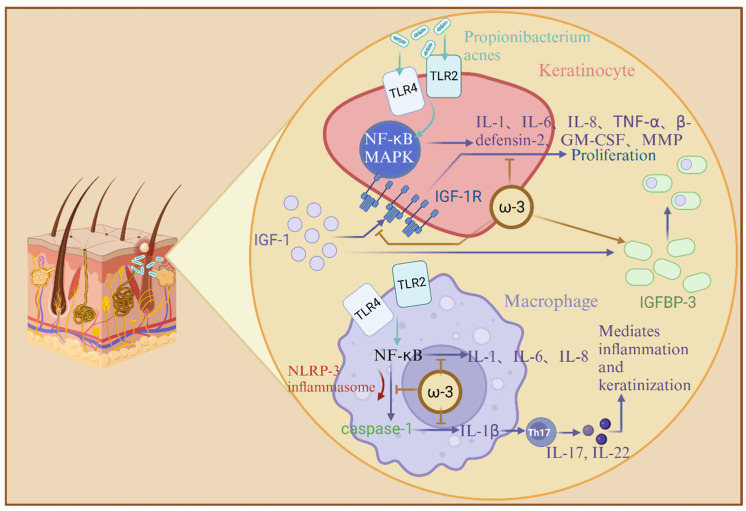
Schematic diagram of the mechanism of omega-3 unsaturated fatty acids affecting acne: TLR-2 and TLR-4 activate the NF-κB and MAPK pathways, producing cytokines such as IL-1, IL-6, IL-8, and TNF-α; omega-3 unsaturated fatty acids increase IGFBP-3, preventing IGF-1 from binding to its receptors; omega-3 unsaturated fatty acids inhibit NF-κB activation; omega-3 unsaturated fatty acids inhibit the activation of NLRP-3 inflammasomes in antigen presenting cells.

*Propionibacterium acnes* can induce keratinocytes to activate TLR-2 and TLR-4, thereby activating the NF-κB and MAPK pathways, which, in turn, leads to the production of interleukin-1 (IL-1), interleukin-6 (IL-6), interleukin-8 (IL-8), tumor necrosis factor-α (TNF-α), human β-defensin-2, granulocyte-macrophage colony stimulating factor (GM-CSF), and matrix metalloproteinase (MMP). Human β-defensin-2 belongs to the family of antimicrobial peptides (i.e., β-defensin) and is involved in the development of acne inflammation. β-defensin induces the release of pro-inflammatory cytokines and chemotaxis of immune-active cells and regulates cell maturation and migration [100]. In addition, omega-3 unsaturated fatty acids inhibit activation of NLRP-3 inflammasomes in antigen presenting cells. Activation of NLRP-3 inflammasomes can enable caspase-1 to be activated, resulting in the release of monocyte-derived interleukin-1β (IL-1β) [101,102]. IL-1β promotes helper T cell 17 (Th17) activation, resulting in IL-17-mediated inflammation and keratinization (Figure 4). This process is regulated by reactive oxygen species (ROS) and proteases [103]. Studies have shown that DHA inhibits the expression of pro-IL-1β and the secretion of mature IL-1β in monocytes [98]. Furthermore, EPA has been shown to inhibit NF-κB activation. After NF-κB is activated, pro-inflammatory cytokines can be transcribed, including IL-1, IL-6, and IL-8. EPA can also inhibit the expression of MMP-9, an endopeptidase that degrades components of the extracellular matrix and amplifies inflammation [104]. Insulin-like growth factor-1 (IGF-1) signaling is one of the most critical pathways in the development of acne, which can induce the expression of pro-inflammatory cytokines (IL-1β, IL-6, IL-8, and TNF-α) and MMP in human sebum cells [105]. Several studies have shown that omega-3 unsaturated fatty acids can lower serum IGF-1 levels and increase insulin-like growth factor binding protein-3 (IGFBP-3), thereby preventing IGF-1 from binding to its receptors [106]. There are many symptoms in skin diseases, and the increased permeability of scaly skin and skin is the first to attract people’s attention to the symptoms caused by a lack of fatty acids, LA, and fatty acids. Its target, PPAR, can restore symptoms after activation, and this target is involved in body activities such as lipid metabolism, inflammation, and keratinocyte differentiation. The author believes that the effect of unsaturated fatty acids on this target should not be limited to the treatment of one disease, especially regarding lipid metabolism and inflammation, which involve many chronic diseases. Therefore, targeting PPAR sites through unsaturated fatty acids can be used as a research direction. As one of the most common skin diseases, acne is a bacterial invasion and an inflammatory reactions. Studies have shown that omega-3 unsaturated fatty acids work on various inflammation pathways. The author believes that in clinical treatment, omega-3 unsaturated fatty acids can be treated with antibacterial drugs to increase the therapeutic effect.

### 3.5. Neurodegenerative Diseases

The most common neurodegenerative diseases are Alzheimer’s disease (AD) and Parkinson’s disease (PD) [107]. Although the pathogenesis and clinical characteristics of AD and PD are different, these diseases share common impairment mechanisms, such as mitochondrial dysfunction [108], neuroinflammation [109], and oxidative stress [110]. AD is characterized by dementia, memory loss, and cognitive decline, which worsen with age [111]. As an answer to the previous question, it has been found that omega-3 unsaturated fatty acids are closely related to these complex diseases. It was found that people with higher DHA intakes have a lower risk of cognitive impairment or AD [112,113]. DHA induces activation of synaptophysin-1, a synaptic vesicle glycoprotein that facilitates synaptogenesis and enhances cognitive performance [114,115]. Mechanistically, synaptophysin-1 mediates neurotransmitter exocytosis, axonal elongation, and synaptic contact maintenance [116,117], with its biosynthesis and subsequent phosphorylation being regulated by brain-derived neurotrophic factor [118,119]. DHA promotes synaptic transmission through brain-derived neurotrophic factor upregulation, thereby establishing a neurotrophic pathway-dependent mechanism underlying synaptophysin-1-mediated neural plasticity [117]. On the other hand, DHA can also inhibit tau phosphorylation and prevent microtubule decomposition and accumulation of neurofibrillary tangles [120]. DHA can also reduce the toxicity of Aβ [121] and apoptosis of neurons [122] by preventing the formation and accumulation of neurons [116,123].

DHA can also regulate the expression of α-synuclein in PD, thereby maintaining synaptic homeostasis and neuronal activity [111]. DHA prevents glial dysfunction [124] in early neurodegenerative diseases by increasing the expression of brain trophic factors, including glially derived neurotrophic factors (GDNFs) [125]. The role of AA in neurodegenerative pathology, such as AD and PD, has not been elucidated. Several studies have shown that a high AA diet in AD mouse models helps to prevent cognitive dysfunction caused by abnormal processing of amyloid precursor protein (APP), thereby reducing the formation of insoluble Aβ in neuritis plaques [126,127]. However, studies have also shown that Aβ production and deposition are increased in mice enriched with an AA diet [128]. It is speculated that the difference may be due to the different doses of AA used.

Studies have suggested that the metabolite of AA, epoxy eicostrienoic acid (EET), can constitute a therapeutic target for PD because they are widely distributed in the brain and shows anti-inflammatory and antioxidant effects [129]. In the Drosophila model of PD, EET increases the expression of antioxidant enzymes and reduces oxidative stress and inflammation [130]. In addition, in vitro studies have found that AA can induce alpha helical assembly of α-synuclein, and neuronal damage can be reduced compared to beta-sheet polymers that have not been treated with AA [131]. Furthermore, α-synuclein is assembled into toxic β-sheet aggregates in PD, and α-helical oligomers in healthy neurons [131]. Unsaturated fatty acids are closely linked to the nervous system and are of great significance to the development and outcome of AD and PD. More intake of omega-3 unsaturated fatty acids can enhance synaptic plasticity, inhibit inflammatory responses, protect cholinergic neurons, and help brain nerve health. The metabolites of omega-6 unsaturated fatty acids have both anti-inflammatory and pro-inflammatory effects, and their effects on AD and PD need more research. Since the inflammation hypothesis is of great significance in the pathogenesis of AD, the author believes that elucidating the regulatory effect of dietary factors on inflammation may help prevent the occurrence of AD, delay the onset of AD, and slow the progress of AD. At the same time, more and more research evidence suggests that other nutrients, such as B vitamins, are related to cognitive impairment in the elderly and may have synergistic effects with omega-3 unsaturated fatty acids [132,133]. Therefore, the author believes that in subsequent research, it is necessary to deeply analyze the mechanism of the effect of the combined supplementation of omega-3 unsaturated fatty acids and different nutrients on cognitive function. Based on the factors and mechanisms of the occurrence and development of cognitive damage, a targeted joint supplementation experiment of omega-3 unsaturated fatty acids and other nutrients is carried out to explore the impact of the combined supplementation of multiple nutrients on the cognitive function of the elderly, and to clarify the role of dietary nutrients in preventing and delaying cognitive impairment in the elderly. In addition, the inability to detect the brain’s sensitivity to unsaturated fatty acids from multiple aspects is a limitation of the current study. In the future, the detection of unsaturated fatty acid genes and the discovery of molecular mechanisms is a new research approach. Based on this, a reasonable dietary intake guide for unsaturated fatty acids will provide new ideas for the research of health care and prevention drugs for the elderly.

### 3.6. Depression

Depression is one of the most common types of mental illnesses in the world, with a prevalence of twice that of men in women [134]. Depression is a multifactorial disease, and heredity and diet are considered to play an important role in its cause and treatment [135]. Some preclinical studies have shown that omega-3 unsaturated fatty acids can act in the hippocampus to prevent or reduce the incidence of depression. Rats who were consuming a high EPA diet for 6 weeks showed increased dopamine and serotonin concentrations in the hippocampus [136], indicating that this omega-3 unsaturated fatty acid prevents depression-like behavior. The offspring of newborn rats who lack omega-3 unsaturated fatty acids in the diet developed nerve damage in the hippocampus and reduced serotonin and norepinephrine levels [137]. The link between the role of omega-3 unsaturated fatty acids on the hypothalamic-pituitary-adrenal (HPA) axis and depression is not clear. However, studies have shown that cortisol levels in patients with depression are negatively correlated with EPA and DHA concentrations in the blood [138]. In addition, in patients diagnosed with depression, increasing dietary EPA intake for 8 weeks can reduce HPA axis activity and improve depression symptoms in these patients [139]. The lack of omega-3 unsaturated fatty acids in the diet also changes the membrane phospholipid composition of the prefrontal cortex (PFC). Experiments have observed that the levels of omega-3 unsaturated fatty acids in PFCs of rats exposed to ALA-deficient diets for a long time were lower. Furthermore, endogenous dopamine levels in PFCs in omega-3 unsaturated fatty acid-deficient animals decreased and serotonin neurotransmission was altered [140], which may indicate that these animals are more likely to develop a depression-like phenotype.

Observations show that omega-6 unsaturated fatty acids are associated with depression because the increase in total levels of omega-6 unsaturated fatty acid-derived endocannabinoids (such as AEA) in the brain impairs serotonin neurotransmission in PFCs and induces a depression-like phenotype in mice [141]. However, there is still controversy about what the relationship between omega-6 unsaturated fatty acids and depression. A study of postmortem human PFC samples found that DHA levels in membrane phospholipids were reduced and AA:DHA ratios were increased [142,143] in patients diagnosed with depression. However, another study was conducted in autopsy PFC samples from males with depressed suicide and did not find any changes in the levels of omega-6 unsaturated fatty acids in PFCs [144]. In addition, in the striatum, data showed that striatum serotonin levels in rats fed a high omega-6 unsaturated fatty acid diet for 8 weeks were reduced [145].

At present, in addition to antidepressant treatment, unsaturated fatty acids have great potential as emerging auxiliary treatment methods for depression. The antidepressant mechanism of omega-3 unsaturated fatty acids involves the hippocampus and PFC. However, there are still some issues that need to be solved worth discussing: the antidepressant effects and mechanisms of EPA and DHA are not exactly the same. In the future, we need to further explore the effects and specific mechanisms of the single component of omega-3 unsaturated fatty acids on the treatment of depression, that is, we need to determine whether EPA or DHA alone is effective, which one is better, or whether the combination of the two is more effective? The antidepressant mechanism of omega-3 unsaturated fatty acids may be very complex, and a large number of basic experiments will be needed in the future to further reveal and provide a theoretical basis for it. In addition, it should be clarified that the effect of omega-3 unsaturated fatty acids on depression is limited to patients with depression who have omega-3 unsaturated fatty acid deficiency, or is effective for ordinary depressed patients. In patients diagnosed with depression, the AA:DHA ratio was found to be increased [142,143], so whether the reduction of omega-6 unsaturated fatty acids/omega-3 unsaturated fatty acid ratio can alleviate the symptoms of depression remains to be further explored. In short, although the mechanism of action of unsaturated fatty acids in patients with depression remains unclear, proper supplementation of omega-3 unsaturated fatty acids can reduce the incidence of depression and reduce the symptoms of depression. The serum omega-3 unsaturated fatty acid level and the ratio of omega-6 unsaturated fatty acids/omega-3 unsaturated fatty acids may predict the severity of depression symptoms and provide a reference for future estimates of the outcome of antidepressant treatment in patients with depression. Table 1 summarizes the roles and underlying mechanisms of omega-3 and omega-6 polyunsaturated fatty acids in the aforementioned pathological conditions.

## 4. Food in Life

Functional foods offer great health benefits to consumers as they can improve general health conditions and reduce the risk of certain diseases.

### 4.1. Unsaturated Fatty Acids in the Diet

As an essential fatty acid that cannot be produced from scratch, humans must obtain omega-3 unsaturated fatty acids through diet, and their most effective source is oily sea fish. Although marine fish, like humans, cannot synthesize these oils themselves, they can accumulate omega-3 unsaturated fatty acids through edible algae and plankton. Although many marine fish are rich in omega-3 unsaturated fatty acids, there are still differences between species. Fish such as mackerel and salmon contain up to 4 g of mixed omega-3 unsaturated fatty acids, while some white fish, such as tilapia, have 10 times less omega-3 unsaturated fatty acids than these marine fish, but have higher omega-6 unsaturated fatty acids. Fish accumulate omega-3 unsaturated fatty acids by eating marine plants that can synthesize these polyunsaturated fatty acids [146]. But if natural algae are replaced by other food sources, such as vegetable diets rich in omega-6 unsaturated fatty acids (such as linoleic acid), the tissue levels of EPA and DHA in fish will drop sharply, just like some commercially farmed fish [147,148]. Omega-3 unsaturated fatty acids are mainly present in esterified form, omega-3 unsaturated fatty acids in fish are usually stored in TG, while 30–65% of krill EPA and DHA are stored in phospholipids [149]. Alpha-linolenic acid (ALA) in omega-3 unsaturated fatty acids can be obtained from plant sources such as flaxseed oil. However, due to its conversion rate limitations, humans must rely primarily on fish rich in omega-3 unsaturated fatty acids to obtain sufficient amounts of EPA and DHA, which are crucial for multi-organ functions, including the nerve and cardiovascular systems.

### 4.2. Conjugated Linoleic Acid

**Figure 5:**
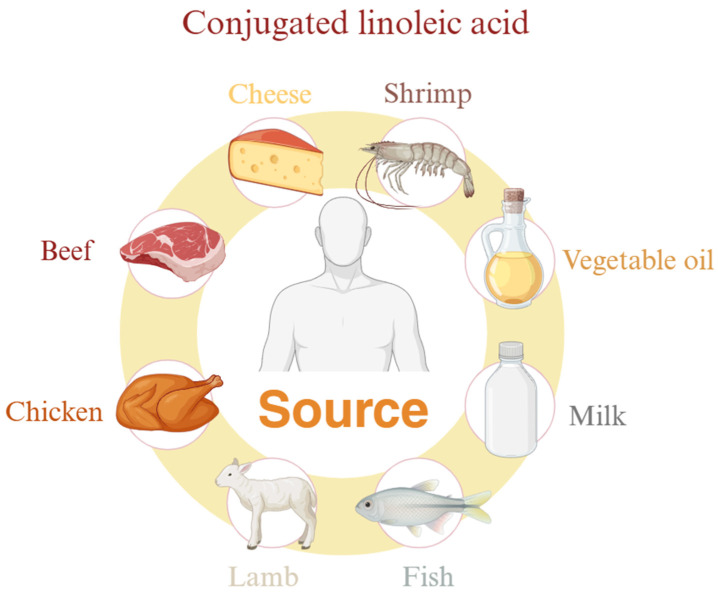
Food sources of CLA.

Conjugated linoleic acid (CLA) has attracted great attention from the scientific community over the past few decades and is considered a food supplement to reduce weight, muscle damage, and inflammatory responses. CLA is a family of unsaturated fatty acids, a mixture of the position of LA and geometric isomers whose conjugated double bonds can be located anywhere in the carbon chain, usually between 8 and 13, and in a cis or trans configuration. They are naturally found in dairy products (milk, cheese, and yogurt) and ruminant meats (beef and lamb) (Figure 5). It is synthesized by rumen bacteria with LA as substrate, but it contains very little. 

CLA has many benefits for the human body, such as the ability to achieve anti-obesity effects by promoting fatty acid oxidation, increasing fat decomposition and energy consumption, and regulating adipocyte metabolism, adipose factors, and cytokines [150,151,152,153]. Furthermore, CLA is the most active antioxidant in cream fat, and its effects may be related to the synergistic effects of other milk components (α-tocopherol, β-carotene, vitamin A and vitamin D3, phospholipids, short-chain saturated fatty acids, vaccine acids, coenzyme Q10, and ether lipids) [154]. In human and animal models, CLA can achieve immunomodulation by regulating lipid metabolism, cell survival, and signal activation [155,156,157,158]. For example, phagocytosis can be improved through the PPAR-γ-dependent pathway and exert its immune stimulation effect [156,159,160]. CLA may also have certain anti-cancer effects, but its exact mechanism is not yet known. This may be related to antioxidant and anti-inflammatory properties and reduced cell proliferation [153,161]. It may also be related to the induction of mitochondrial apoptosis pathways [162]. Although CLA has many beneficial effects, such as anti-obesity, anti-inflammatory, antioxidant, immune regulation, anti-cancer, and anti-atherosclerotic effects, it may also cause some health problems. For example, CLA supplements may aggravate insulin resistance in patients with diabetes or metabolic syndrome [163]. In addition, it may also affect the intestinal tract, fertility [164], and vitamin A metabolism [165].

CLA has many benefits for human health and is universally safe for humans [166]. At present, the conjugated linoleic acids that are industrially synthesized and commercially available are mainly mixtures of t10c12-CLA and c9t11-CLA. Various studies have proved that CLA has played a certain role in the treatment of anti-cancer, anti-inflammatory, and metabolic diseases, but according to existing clinical studies and animal experiments, it can be seen that the effects of different conjugated linoleic acid isomers also have different deviations. Therefore, the author believes that it is urgent to study the functional mechanism of a single CLA isomer from the molecular level. CLA also plays an important role in improving blood lipid levels, regulating fat metabolism [167], inhibiting cancer development [168], and immunomodulation [156]. However, risks are still controversial in regulating the sensitivity of blood sugar and insulin and the impact of fertility. It is necessary to further investigate the health status of different types of organisms after application. In addition, the author believes that how to produce high-purity CLA through industry and how to prove the mechanism of CLA’s actual role in diseases may become a hot topic in future research.

## 5. Discussion

In recent years, unsaturated fatty acids have attracted much attention in diet therapy and chronic disease treatment. In terms of cardiovascular disease, omega-3 unsaturated fatty acids reduce triglyceride levels through various mechanisms, thereby reducing the risk of cardiovascular dysfunction and atherosclerosis. Excessive levels of omega-6 unsaturated fatty acids may increase the production of prostaglandin 2 and leukotriene 4, thereby exerting a pro-inflammatory effect. In the field of diabetes, elevated triglycerides not only increase the risk of cardiovascular disease, but are also one of the main risk factors for type 2 diabetes. omega-3 unsaturated fatty acids reduce the risk of type 2 diabetes by reducing triglyceride levels [53] and improving insulin resistance. In the field of cancer, omega-3 unsaturated fatty acids are also closely related to their pathogenesis [169], such as DHA and EPA, which show apoptosis-induced effects on a variety of cancer cells [84], providing new auxiliary means for cancer treatment. At the same time, they may also increase the sensitivity of tumors to radiotherapy and protect skin tissues in non-radio areas, thereby further enhancing the efficacy of cancer. In the field of skin diseases, omega-6 unsaturated fatty acids (such as LA) maintain skin barrier function by activating PPAR-α, while omega-3 unsaturated fatty acids reduce acne inflammation by inhibiting TLR signaling and NLRP-3 inflammasomes, providing a molecular basis for targeted treatment of skin diseases. In the field of neurodegenerative diseases, DHA delays the progression of Alzheimer’s disease by inhibiting Aβ deposition, reducing tau protein phosphorylation, and enhancing synaptic plasticity; omega-6 unsaturated fatty acid metabolites (such as EET) may improve Parkinson’s disease symptoms through antioxidant and anti-inflammatory effects. The dynamic balance of the two in the brain may be the key to neuroprotection. In the field of depression, omega-3 unsaturated fatty acids relieve depression symptoms by regulating hippocampal monoamine neurotransmitters (such as 5-HT, DA) and inhibiting hyperactivation of the HPA axis, while metabolic imbalance of omega-6 unsaturated fatty acids (such as an increase in AA/DHA ratio) may aggravate neuroinflammatory and mood disorders.

Despite all the benefits of unsaturated fatty acids, their possible dissipative consequences, such as lipid peroxidation products, should not be overlooked. Lipid peroxidation products critically influence chronic disease pathogenesis through membrane destabilization, neurotoxicity, and metabolic dysregulation. The structural instability of unsaturated fatty acids facilitates peroxidation, generating lipid hydroperoxides (LOOHs) as primary non-radical intermediates. These LOOHs decompose into reactive aldehydes (e.g., malondialdehyde, 4-hydroxynonenal), ketones, and epoxides, which disrupt intracellular membranes and drive neurodegenerative, gastric, and nutritional pathologies [170]. Peroxyl (LOO•) and alkoxyl (LO•) radicals, formed via metal ion-mediated LOOH decomposition, propagate oxidative cascades through the Russell mechanism, yielding cytotoxic lipid fragments and singlet oxygen (1O2), exacerbating oxidative stress, apoptosis, and tissue damage [171]. Notably, phospholipid hydroperoxides impair membrane fluidity and signaling, while cholesterol hydroperoxides in oxidized LDL promote atherosclerotic plaque formation [171]. The accumulation of 4-hydroxynonenal (4-HNE) and malondialdehyde (MDA) directly contributes to neurodegenerative disorders by covalently modifying neuronal proteins and nucleic acids, impairing function, and accelerating amyloid-beta aggregation in Alzheimer’s disease [171]. These mechanisms highlight the dual role of lipid oxidation in membrane destabilization and chronic disease progression, necessitating advanced analytical approaches to delineate therapeutic targets [170,172]. Mass Spectrometry and Nuclear Magnetic Resonance Spectroscopy will undoubtedly continue to be developed as major structural and analytical tools in the field of lipid hydroperoxides research [173].

Although the role of omega-3 and omega-6 unsaturated fatty acids in chronic diseases has been widely studied, there are still many unknown areas to be explored. For example, omega-6 unsaturated fatty acids may show positive effects in some cases, but their specific mechanisms are not fully understood. In addition, different populations may have different responses to unsaturated fatty acids, which may be related to various factors such as genetics, lifestyle, and environmental factors. Therefore, future research needs to explore more in-depth how these factors affect the effects of unsaturated fatty acids. In terms of the ratio of omega-3 and omega-6 unsaturated fatty acids, although the importance of this ratio has been continuously emphasized, there is no consensus on the specific optimal ratio. However, an interesting study says that in breastfeeding women, supplementation with omega-3 unsaturated fatty acids through the addition of a combination of concentrated margarine and canola oil increased the ALA content of breast milk and produced the most favorable LA-ALA ratios for the synthesis of long-chain unsaturated fatty acids [174]. Different diseases may require differentiated regulation [175], while current dietary guidelines are mostly based on epidemiological data and lack precise molecular mechanism support. With an in-depth understanding of the mechanism of action of omega-3 and omega-6 unsaturated fatty acids in chronic diseases, future research will pay more attention to their specific application effects in different populations. For example, for people with a high risk of cardiovascular disease and diabetes, developing functional foods or drugs rich in omega-3 unsaturated fatty acids may become an effective prevention and treatment strategy.

## 6. Conclusions

**Figure 6:**
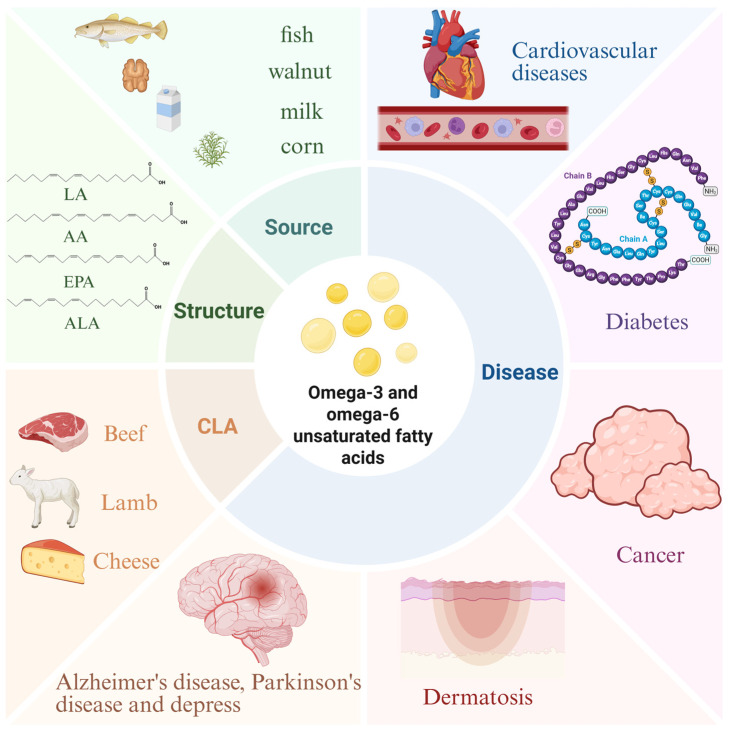
Overview of this article.

This article elaborates on the structural properties, metabolic pathways, and dietary sources of omega-3 and omega-6 unsaturated fatty acids in detail, and focuses on their mechanisms of action in a variety of chronic diseases. Omega-3 unsaturated fatty acids have been found to have multiple benefits, such as reducing the risk of cardiovascular disease [176], improving insulin resistance [177], reducing inflammation, and promoting brain development. In contrast, the role of omega-6 unsaturated fatty acids is more complicated. Although it is an essential fatty acid in the human body, excessive intake may increase the risk of cardiovascular disease [44], although this view remains controversial. As for the dose-response effect of fatty acids, it has been shown that there is a stronger, approximately linear, dose-response relationship with omega-3 unsaturated fatty acids among hyperlipidemic and hypertensive populations, implying that this population may be more sensitive to the beneficial effects of omega-3 unsaturated fatty acid intake on blood pressure reduction [178]. However, there are also data that also suggest that omega-3 unsaturated fatty acid intake above the recommended intake of 3 g/d is not associated with additional benefits, especially in the subgroup with normal blood pressure [178]. The dose-response relationship linking omega-3 unsaturated fatty acid intake to cognitive function is also of interest. It was found that the beneficial effects on executive function tended to increase with intakes of more than 500 mg of omega-3 unsaturated fatty acids and up to 420 mg of EPA per day during the first 12 months. In contrast, after 12 months of intervention, a downward curve was observed at doses of EPA above 420 mg/d. Moreover, these trends were more pronounced in areas where blood levels of DHA and EPA were not very low [178]. This article also emphasizes that the ratio between omega-3 and omega-6 unsaturated fatty acids may be more important than the absolute intake of both. In addition, by adjusting dietary structure and improving lifestyle, people can also better utilize the benefits of these unsaturated fatty acids to maintain their health. Figure 6 provides a comprehensive synthesis of the key aspects discussed in this review.

## Figures and Tables

**Table 1:** Roles and Mechanisms of Omega-3 and Omega-6 polyunsaturated Fatty Acids in Cardiovascular Diseases, Diabetes, Cancer, Dermatological Disorders, Neurodegenerative Diseases, and Depression.

Diseases	Omega-3 Unsaturated Fatty Acids	Omega-6 Unsaturated Fatty Acids
Cardiovascular Disease	Omega-3 unsaturated fatty acids, including eicosapentaenoic acid (EPA) and docosahexaenoic acid (DHA), exert cardioprotective effects primarily by reducing triglyceride (TG) levels and improving endothelial function. These effects are mediated through multiple pathways: inhibition of hepatic diacylglycerol acyltransferase (DGAT) and phosphatidic acid phosphatase, key enzymes in TG synthesis, thereby suppressing hepatic triglyceride production and VLDL secretion. Omega-3 unsaturated fatty acids enhance fatty acid β-oxidation while reducing substrate availability for TG formation via acyl-CoA synthetase inhibition. Additionally, they modulate lipoprotein metabolism by lowering apolipoprotein C3 levels and activating lipoprotein lipase (LPL), facilitating clearance of triglyceride-rich lipoproteins. EPA, as a potent peroxisome proliferator-activated receptor (PPAR) agonist, further enhances lipid metabolism pathways. Beyond lipid regulation, omega-3 unsaturated fatty acids improve endothelial function by promoting nitric oxide-dependent vasodilation and attenuating pro-inflammatory cytokine production, thereby mitigating vascular inflammation and endothelial dysfunction, critical factors in atherosclerosis progression.	Omega-6 unsaturated fatty acids, such as arachidonic acid (AA), demonstrate ambivalent cardiovascular effects. While theoretical concerns exist regarding their pro-inflammatory potential via increased prostaglandin E2 (PGE2) and leukotriene B4 synthesis, observational studies suggest an inverse correlation between ω-6 intake and cardiovascular disease (CVD) risk. Current evidence emphasizes the importance of the ω-6/ω-3 ratio over absolute intake levels. However, ω-6 derivatives like AA may enhance lipoprotein oxidation susceptibility, potentially exacerbating atherogenesis.
Diabetes	Omega-3 unsaturated fatty acids, particularly those derived from marine sources, are associated with a reduced risk of type 2 diabetes mellitus through multifaceted mechanisms. By lowering systemic triglyceride (TG) levels, omega-3 fatty acids mitigate the accumulation of free fatty acids, diacylglycerol (DAG), and fatty acyl-CoA, thereby reducing mitochondrial dysfunction, oxidative stress, and subsequent insulin resistance (IR). These effects are mediated via activation of peroxisome proliferator-activated receptors (PPAR-α and PPAR-γ), which enhance insulin sensitivity, suppress pro-inflammatory pathways, and promote fatty acid β-oxidation. Additionally, omega-3 fatty acids improve mitochondrial and endoplasmic reticulum function, reducing ectopic lipid deposition in muscles and enhancing cellular energy metabolism. Their ability to counteract IR and preserve β-cell function further underscores their therapeutic potential in type 2 diabetes mellitus prevention and management.	The relationship between omega-6 unsaturated fatty acids, particularly linoleic acid (LA), and type 2 diabetes mellitus risk remains complex and context-dependent. Epidemiological evidence suggests that higher dietary LA intake and elevated circulating LA levels correlate with reduced type 2 diabetes mellitus incidence, potentially through mechanisms involving improved cellular membrane fluidity. Enhanced membrane properties may facilitate insulin receptor binding, GLUT transporter translocation, and intracellular signaling, collectively boosting insulin sensitivity. LA also modulates lipid metabolism by regulating sterol regulatory element-binding transcription factor 1 (SREBP1), balancing hepatic fatty acid synthesis and oxidation, which may reduce hepatic steatosis. Furthermore, LA-rich diets are linked to favorable abdominal fat distribution and metabolic improvements. However, the precise biological pathways underlying these associations require further elucidation, and the overall impact of omega-6 fatty acids may depend on their ratio to omega-3 intake, highlighting the need for balanced dietary strategies.
Cancer	Omega-3 unsaturated fatty acids, particularly eicosapentaenoic acid (EPA) and docosahexaenoic acid (DHA), exhibit chemopreventive and antitumor properties across multiple cancer types, including leukemia, breast, colon, prostate, and melanoma. These effects are mediated through modulation of inflammatory pathways, suppression of pro-tumorigenic mediators, and induction of apoptotic mechanisms. Omega-3 fatty acids reduce pro-inflammatory eicosanoid production by competitively inhibiting cyclooxygenase and lipoxygenase pathways, thereby attenuating inflammation-driven carcinogenesis. They promote mitochondrial dysfunction in cancer cells by increasing reactive oxygen species (ROS) generation, altering mitochondrial membrane potential, and activating caspase-dependent apoptosis via caspase-3, caspase-8, and caspase-9. DHA specifically induces G1-phase cell cycle arrest in ovarian cancer cells by downregulating cyclin-dependent kinases (CDK4, CDK6), cyclin D1, and anti-apoptotic protein Mcl-1. Additionally, omega-3 fatty acids inhibit tumor angiogenesis by reducing pro-angiogenic arachidonic acid (AA)-derived prostaglandin E2 (PGE2) while increasing anti-angiogenic resolvins and neuroprotectins. Their metabolites, such as resolvin D1 (RvD1), suppress metastatic progression by downregulating chemokine receptor CXCR4 expression, impairing cancer cell migration and invasion. Omega-3 fatty acids also counteract inducible nitric oxide synthase (iNOS)-mediated nitric oxide (NO) production, a key driver of tumor progression, through anti-inflammatory and antioxidant mechanisms.	The role of omega-6 unsaturated fatty acids in cancer is dualistic, with both pro- and anti-tumorigenic effects reported. High omega-6 intake, particularly linoleic acid (LA), may promote carcinogenesis by elevating pro-inflammatory eicosanoids such as prostaglandin E2 (PGE2), which enhance tumor cell proliferation, angiogenesis, and immune evasion. However, omega-6 derivatives like arachidonic acid (AA) can also exert antitumor effects by inducing mitochondrial ROS overproduction, triggering oxidative damage, and impairing cancer cell survival. The omega-6/omega-3 ratio critically influences cancer outcomes: a lower ratio upregulates tumor suppressor SMAR1 and p21 protein expression, inhibiting cell cycle progression, while reducing oncogenic MARBP Cux/CDP activity. Despite potential pro-inflammatory effects, epidemiological and experimental data suggest that balanced omega-6 intake may improve membrane fluidity and cellular signaling, indirectly modulating tumorigenic pathways. The complexity of omega-6 effects underscores the importance of dietary context and metabolic interactions in determining their net impact on cancer progression.
Skin Diseases	Omega-3 unsaturated fatty acids, notably docosahexaenoic acid (DHA) and eicosapentaenoic acid (EPA), exhibit potent anti-inflammatory and therapeutic effects in inflammatory skin disorders such as acne vulgaris. These fatty acids attenuate acne-associated inflammation by targeting Toll-like receptor (TLR) signaling pathways, specifically inhibiting TLR-1 and TLR-2 dimerization, which is critical for Cutibacterium acnes-induced hyperproliferation of keratinocytes and macrophage-driven cytokine production. DHA and EPA suppress the activation of NF-κB and MAPK pathways, reducing the expression of pro-inflammatory mediators, including interleukin-1β (IL-1β), IL-6, IL-8, TNF-α, and matrix metalloproteinase-9 (MMP-9). Additionally, omega-3 fatty acids inhibit NLRP3 inflammasome activation in antigen-presenting cells, thereby blocking caspase-1-dependent IL-1β maturation and secretion. They further modulate insulin-like growth factor-1 (IGF-1) signaling by lowering serum IGF-1 levels and elevating insulin-like growth factor-binding protein-3 (IGFBP-3), which disrupts IGF-1 receptor binding and downstream pro-inflammatory cytokine release. By mitigating oxidative stress and IL-17-mediated keratinization, omega-3 fatty acids counteract C. acnes-triggered Th17 activation and extracellular matrix degradation, offering a multi-targeted approach to acne management.	Linoleic acid (LA), a predominant omega-6 unsaturated fatty acid, plays a pivotal role in maintaining epidermal barrier integrity and function. LA deficiency is linked to impaired skin permeability, characterized by scaly dermatitis and increased transepidermal water loss (TEWL), which can be reversed through topical LA application. Mechanistically, LA activates peroxisome proliferator-activated receptors (PPAR-α), enhancing lipid metabolism, keratinocyte differentiation, and barrier homeostasis. It also serves as a substrate for β-oxidation, fueling sebaceous gland synthesis of barrier-associated lipids such as squalene and wax esters. While LA is essential for skin structural integrity, its role in acne pathogenesis remains indirect. By improving membrane fluidity and modulating inflammatory pathways, LA may indirectly influence acne-related processes; however, excessive omega-6 intake may alter eicosanoid profiles, potentially exacerbating inflammation. The balance between omega-6 and omega-3 fatty acids is critical, as their interplay regulates both barrier function and inflammatory responses in cutaneous health.
Neurodegenerative Diseases	Omega-3 unsaturated fatty acids, particularly docosahexaenoic acid (DHA), demonstrate neuroprotective effects in Alzheimer’s disease (AD) and Parkinson’s disease (PD) by targeting shared pathological mechanisms such as mitochondrial dysfunction, neuroinflammation, and oxidative stress. DHA enhances synaptic plasticity and cognitive function through activation of synaptophysin-1, a protein critical for neurotransmitter release, axonal elongation, and synaptic maintenance. This process is facilitated by DHA-mediated upregulation of brain-derived neurotrophic factors, which promote synaptogenesis and neuronal survival. Additionally, DHA attenuates AD progression by inhibiting hyperphosphorylation of tau protein, thereby preventing microtubule destabilization and neurofibrillary tangle formation, and by reducing amyloid-β (Aβ) toxicity through suppression of Aβ production and aggregation. In PD models, DHA modulates α-synuclein expression, maintaining synaptic homeostasis and neuronal activity, while upregulating neuroprotective factors like glial cell line-derived neurotrophic factor to counteract early glial dysfunction. These multifaceted actions underscore DHA’s role in mitigating neurodegeneration via anti-inflammatory, antioxidant, and pro-synaptic mechanisms.	The impact of omega-6 unsaturated fatty acids, such as arachidonic acid (AA), on neurodegenerative diseases remains complex and context-dependent. AA metabolites, including epoxyeicosatrienoic acids (EETs), exhibit neuroprotective potential in PD by reducing oxidative stress and neuroinflammation, as evidenced by enhanced antioxidant enzyme expression and attenuated α-synuclein toxicity through promotion of its non-pathogenic α-helical oligomerization. However, conflicting data exist regarding AA’s role in AD: some studies suggest it may reduce insoluble Aβ plaque formation by modulating amyloid precursor protein (APP) processing, while others report increased Aβ deposition, potentially linked to dosage-dependent effects. AA’s dual influence—balancing anti-inflammatory EET production against pro-inflammatory eicosanoid pathways—highlights its nuanced role in neurodegeneration. Current evidence emphasizes the need to further investigate the interplay between omega-6 metabolites, neuroinflammatory cascades, and protein aggregation dynamics, particularly in the context of dietary ratios to omega-3 fatty acids, which collectively shape neuronal resilience and disease progression.
Depression	Omega-3 unsaturated fatty acids, particularly eicosapentaenoic acid (EPA) and docosahexaenoic acid (DHA), exert antidepressant effects through modulation of neurotransmitter homeostasis, neuroplasticity, and hypothalamic-pituitary-adrenal (HPA) axis activity. EPA enhances hippocampal dopamine and serotonin concentrations, counteracting depressive-like behaviors in preclinical models, while dietary deficiency of omega-3 fatty acids disrupts prefrontal cortical membrane phospholipid composition, reducing endogenous dopamine and altering serotonergic neurotransmission. Omega-3 supplementation attenuates HPA axis hyperactivity, as evidenced by reduced cortisol levels correlating with elevated blood EPA/DHA concentrations in depressed individuals. These fatty acids also preserve synaptic plasticity by maintaining prefrontal cortical lipid architecture, thereby mitigating vulnerability to depression. DHA further stabilizes neuronal membrane fluidity and supports neurotrophic signaling, which collectively enhances resilience to stress-induced neurochemical imbalances.	The role of omega-6 unsaturated fatty acids in depression is marked by conflicting evidence, reflecting their dual influence on neuroinflammatory and endocannabinoid pathways. Elevated omega-6-derived endocannabinoids, such as anandamide (AEA), impair serotonergic neurotransmission in the PFC and induce depressive-like phenotypes in animal models. Postmortem studies in depressed individuals reveal altered membrane phospholipid profiles, including reduced DHA and elevated arachidonic acid (AA)-to-DHA ratios, suggesting a potential dysregulation in fatty acid metabolism. However, inconsistencies exist, as some human and rodent studies report no significant changes in prefrontal cortical omega-6 levels despite depressive pathology. High dietary omega-6 intake may reduce striatal serotonin, exacerbating mood dysregulation, yet the mechanistic interplay between omega-6 metabolites, neuroinflammation, and monoaminergic systems remains poorly defined. The balance between omega-6 and omega-3 fatty acids appears critical, with disproportionate omega-6 intake potentially disrupting neural lipid signaling and amplifying depressive risk through pro-inflammatory eicosanoid pathways.

## Data Availability

No new data were created or analyzed in this study.

## Abbreviations

The following abbreviations are used in this manuscript:

PUFAs	Polyunsaturated fatty acids
AD	Alzheimer’s disease
PD	Parkinson’s disease
ALA	α-Linolenic acid
SDA	Stearidonic acid
EPA	Eicosapentaenoic acid
DPA	Docosapentaenoic acid
DHA	Docosahexaenoic acid
LA	Linoleic acid
AA/ARA	Arachidonic acid
PL	Phospholipids
CE	Cholesterol ester
PLA2	Phospholipase A2
PC	Phosphatidyl choline
FADS1	Fatty acid desaturase 1
ELOVL	Elongase of very-long chain fatty acids
FADS2	Fatty acid desaturase 2
TGRL	Triglyceride rich lipoproteins
DGAT	Diacylglycerol acyltransferase
LPL	Lipoprotein lipase
PPAR	Peroxisome proliferators-activated receptor
FFA	Free fatty acids
HSL	Hormone-sensitive triglyceride lipase
TG	Triglyceride
ROS	Reactive oxygen species
SREBP1	Sterol regulatory element-binding protein-1
NO	Nitric Oxide
mtNOS	Mitochondrial nitric oxide synthase
iNOS	Inducible nitric oxide synthase
F4-NeuroPs	F4-Neuroprostaglandins
PGE2	Prostaglandin 2
RvD1	Resolvin D1
TEWL	Trans epidermal water loss
IL-1	Interleukin-1
IL-6	Interleukin-6
IL-8	Interleukin-8
TNF-α	Tumor necrosis factor-α
GM-CSF	Granulocyte-Macrophage Colony-Stimulating Factor
MMP	Matrix metalloproteinase
IGF-1	Insulin-like growth factor 1
IGFBP-3	Insulin-like growth factor binding protein-3
GDNF	Glial cell derived neurotrophie factor
APP	Amyloid precursor protein
EET	Epoxyeicosatrienoic acid
HPA	Hypothalamic–pituitary–adrenal axis
PFC	Prefrontal cortex
CLA	Conjugated linoleic acid
LPC	Lysophosphatidyl choline
GLA	Gamma linolenic Acid
